# nES GEMMA Analysis of Lectins and Their Interactions with Glycoproteins – Separation, Detection, and Sampling of Noncovalent Biospecific Complexes

**DOI:** 10.1007/s13361-016-1483-0

**Published:** 2016-09-19

**Authors:** Nicole Y. Engel, Victor U. Weiss, Martina Marchetti-Deschmann, Günter Allmaier

**Affiliations:** Institute of Chemical Technologies and Analytics, TU Wien (Vienna University of Technology), Getreidemarkt 9/164-IAC, A-1060 Vienna, Austria

**Keywords:** Lectin, Glycoprotein, nES GEMMA, CE-on-a-chip, Electrophoresis

## Abstract

In order to better understand biological events, lectin–glycoprotein interactions are of interest. The possibility to gather more information than the mere positive or negative response for interactions brought mass spectrometry into the center of many research fields. The presented work shows the potential of a nano-electrospray gas-phase electrophoretic mobility molecular analyzer (nES GEMMA) to detect weak, noncovalent, biospecific interactions besides still unbound glycoproteins and unreacted lectins without prior liquid phase separation. First results for *Sambucus nigra* agglutinin, concanavalin A, and wheat germ agglutinin and their retained noncovalent interactions with glycoproteins in the gas phase are presented. Electrophoretic mobility diameters (EMDs) were obtained by nES GEMMA for all interaction partners correlating very well with molecular masses determined by matrix-assisted laser desorption/ionization mass spectrometry (MALDI-MS) of the individual molecules. Moreover, EMDs measured for the lectin–glycoprotein complexes were in good accordance with theoretically calculated mass values. Special focus was laid on complex formation for different lectin concentrations and binding specificities to evaluate the method with respect to results obtained in the liquid phase. The latter was addressed by capillary electrophoresis on-a-chip (CE-on-a-chip). Of exceptional interest was the fact that the formed complexes could be sampled according to their size onto nitrocellulose membranes after gas-phase separation. Subsequent immunological investigation further proved that the collected complex actually retained its native structure throughout nES GEMMA analysis and sampling.

**Electronic supplementary material:**

The online version of this article (doi:10.1007/s13361-016-1483-0) contains supplementary material, which is available to authorized users.

**Graphical Abstract:**
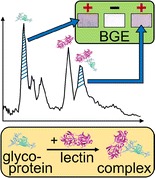
ᅟ

## Introduction

In recent years, the analyses of a variety of macromolecules (e.g. DNA [1], proteins [2–5], polymers [6–8], viruses and virus-like-particles [5, 9, 10], gold nanoparticles [11–13]) have shown the broad applicability of nano-electrospray gas-phase electrophoretic mobility molecular analyzer (nES GEMMA). Thus, this method is used with increasing interest for size-determination of particles ranging from small analytes of only a few nm in size up to particles of several hundred nm. Characterizations with nES GEMMA are generally independent of the analyzed particle type and chemical composition, which makes the method very versatile.

As previously described by Kaufman in detail [3], nES GEMMA separates analytes according to their electrophoretic mobility diameter (EMD) in the gas phase, which can directly be correlated to the dry particle diameters in the nm range. Consequently, the molecular weights can be calculated by application of a correlation derived from respective standard compounds [3, 4]. In brief, multiply charged droplets produced in cone jet mode in the nES unit are dried and simultaneously charge-reduced in a bipolar atmosphere (induced by a ^210^Po source) and subsequently introduced into the nano differential mobility analyzer (nDMA). Dominantly singly charged analytes with a certain EMD can exit the nDMA at a particular applied voltage. For detection, the so obtained monodisperse aerosol is directed into a condensation particle counter (CPC), in which supersaturated n-butanol vapor condenses onto the particles. Following nucleation, single particles can be counted by laser light scattering yielding e.g., a number-based particle concentration.

nES GEMMA also allows a size-selective collection of analytes after gas-phase separation for consecutive investigations like microscopic measurements (transmission electron microscopy, TEM; atomic-force microscopy, AFM) or a biological test as an immunologic assay [14–16]. For this purpose, the CPC is replaced by an electrostatic nanoparticle sampler (ENAS). It consists of an electrically grounded sampling chamber that features an electrode in its bottom center. By application of a negative voltage to this electrode, positively charged particles coming from the nDMA are attracted. Consequently, they are sampled onto a substrate (e.g., TEM grid, freshly cleaved mica plate or nitrocellulose (NC) membrane) mounted on top of the electrode. The deposition rate is affected by the flow rate, with which the analytes enter the sampling chamber, by the applied voltage as well as by the particle concentration and charge.

Operating at ambient pressure and with nondenaturing electrolyte solutions, nES GEMMA has proven its strength to preserve noncovalent interactions [5, 17–21]. Therefore, nES GEMMA can be considered an effective technique to study even very fragile biocomplexes like lectin–glycoprotein. Lectins have become a major tool in the fields of glycomics and are applied in many methods for a specific glycoprotein enrichment, glycan characterization or targeted glycoprotein detection. Some of the most commonly used lectins are *Sambucus nigra* agglutinin (SNA), wheat germ agglutinin (WGA), and concanavalin A (ConA), with varying specificities towards different oligosaccharide structures. SNA, a lectin isolated from elder, consists of two subunits, A and B, linked by disulfide bridges: the A subunit compromises a N-glycosidase activity, whereas the B subunit is responsible for sugar recognition and binding. The lectin specifically recognizes Neu5Acα(2,6)Gal/GalNAc, sialic acids (N-acetylneuraminic acid Neu5Ac) α-glycosidically linked to galactose (Gal), or N-acetylgalactosamine (GalNAc). It features at least two saccharide-binding sites per B subunit [22]. In comparison, the 36 kDa homodimeric WGA preferably binds to terminal N-acetyl-D-glucosamine (GlcNAc) and its β(1,4)-linked oligomers, as well as to Neu5Ac based on its structural similarity towards GlcNAc. WGA, a plant lectin enriched in the seeds of *Triticum vulgaris*, exhibits four sugar binding sites per monomer [23]. The dimeric form is stabilized by ion pairs, several strong H-bonds, and numerous van der Waals’ contacts. The third lectin, ConA, isolated from jack bean (*Canavalia ensiformis*), exists as an oligomer of identical 26 kDa subunits (the exact composition is pH-dependent, see Results and Discussion). It provides one carbohydrate binding site per monomer, which is like the WGA dimer noncovalently linked. ConA specifically binds to mannose (Man) residues as found in the core structure of all N-glycans (Man-α(1,3)[Man-α(1,6)]Man), as well as in high-mannose and hybrid type N-glycans [24, 25].

In the present study, those three lectins were used to analyze their interactions with glycoproteins exhibiting varying glycosylation patterns and degrees for the first time with nES GEMMA. The instrument’s advantage of keeping fragile noncovalent biocomplexes intact allowed the separation and detection of the lectin–glycoprotein complexes. It even enabled an investigation of the lectins’ binding specificities towards the different applied glycoproteins transferrin (Tf), antitrypsin (A1AT), and acid glycoprotein (AGP), especially in comparison to a nonglycosylated negative control β-galactosidase (β-Gal). The chosen set of glycoproteins differed significantly in size, glycosylation degree, and glycosylation pattern (Table 1): Tf, the biggest of the applied glycoproteins in size, featured the lowest glycosylation content with one O-glycan, two N-glycans, and low degree of sialylation [26]. The smaller A1AT exhibited one additional N-glycosylation site and higher degree of sialylation [28]. AGP was the smallest applied glycoprotein with the highest glycan content (five N-glycans) and the highest number of sialic acid residues attached [30].

**Table 1:** Analysis of Tf [26, 27], A1AT [28, 29], AGP [30], β-Gal [31, 32], and SNA [22, 33] by MALDI-MS and nES GEMMA

Protein	Approx. N-glycosylation (w/w %)^a^	N-glycosylation sites^a^	MALDI-MS MW_lit_ (kDa)^a^	MALDI-MS MW_exp_ (kDa)^b^	nES GEMMA EMD_exp_ (nm)^b^	nES GEMMA MW_exp_ (kDa)^c^	nES GEMMA FWHM (nm)^d^
Tf	6	Asn^413^, Asn^611^	80	**79.1** ± 0.1	**7.69** ± 0.04	83.4 ± 1.1	0.31 ± 0.01
A1AT	13	Asn^46^, Asn^83^, Asn^247^		**34.4** ± 0.6	**5.81** ± 0.02	37.7 ± 0.5	0.34 ± 0.01
			51	50.8 ± 0.3	6.58 ± 0.07	53.6 ± 1.6	
AGP	37	Asn^16^, Asn^39^, Asn^76^, Asn^86^, Asn^118^	33.8	**31.2** ± 0.5	**5.59** ± 0.05	33.8 ± 0.9	0.34 ± 0.02
			-	45.5 ± 0.3	6.62 ± 0.05	54.5 ± 1.1	
			-	76.0 ± 0.5	7.83 ± 0.04	87.9 ± 1.1	
β-Gal	0	-	116.3	**116.4** ± 0.1	9.35 ± 0.00	147.2 ± 0.0	
			-	Not detectable	**13.35** ± 0.06	429.4 ± 5.7	0.45 ± 0.06
SNA-I^e^ [A-s-s-B]_2_	5	8 putative	A: 33 ^f)^ B: 35^f)^	**130.1** ± 0.7	**9.40** ± 0.09	149.6 ± 4.4	0.53 ± 0.10
SNA-I^e^ [A-s-s-B]_4_	10	16 putative	-	Not detectable	11.66 ± 0.12	284.7 ± 8.6	

^a^ Values according to references

^b^ Dominating (glyco)protein species in bold

^c^ Values calculated according to [4]

^d^ Calculated after normalization to most abundant peak

^e^ A and B represent the subunits of SNA, -s-s- a disulfide bond, and [ ]_2/4_ a dimeric/tetrameric complex

^f^ Determined by SDS-PAGE under reducing conditions

It was found that nES GEMMA is a straight-forward method with simplified data interpretation due to charge-reduction to singly charged species compared with ESI mass spectra. Biospecific complexes were detected and, furthermore, sampled onto a NC membrane after gas-phase size-separation in the nDMA for analysis with an immunoassay. The transfer of intact noncovalent complexes to the gas phase was additionally underscored by comparing gained nES GEMMA data with theoretical estimated values based on mass calculations. For several lectins and glycoproteins, molecular masses were measured by matrix-assisted laser desorption/ionization time-of-flight MS (MALDI-TOF-MS) in linear mode. They were in good agreement compared with nES GEMMA-based results demonstrating the applicability of this approach. Owing to the weak interactions, the molecular masses of the biospecific complexes were only determined by nES GEMMA. Lectin–glycoprotein complexes at 10.85 nm diameter (229 kDa) were detected for Tf-SNA and discussed in detail. nES GEMMA-based molecular mass values correlated well with the theoretically calculated masses of the biospecific complexes. Finally, the results of the binding experiments were further confirmed by capillary electrophoresis on a chip (CE-on-a-chip) with laser-induced fluorescence (LIF) detection.

## Experimental

### Materials

Ammonium acetate (NH_4_OAc, ≥99.99%), Tween 20 (bioxtra grade), *N,N*-dimethylformamide, trifluoroacetic acid (TFA, ≥99%), sinapic acid (SA, ≥98%), alkaline phosphatase linked antibody (goat, anti-rabbit immunoglobulin), anti-α_1_-antitrypsin antibody (rabbit), and ammonium hydroxide (28.2% ammonia in water) were purchased from Sigma-Aldrich (St. Louis, MO, USA), as were human serum Tf (≥98%), bovine AGP (99%), human A1AT (salt free, lyophilized powder), and β-Gal (lyophilized powder). Lectins SNA, ConA, and WGA were from Vector Laboratories (Burlingame, CA, USA). Sodium chloride (NaCl, ≥99.5%), sodium hydroxide (≥99%), as well as acetonitrile (ACN), hydrochloric acid, magnesium chloride hexahydrate, sodium hydrogen carbonate, tris(hydroxymethyl)aminoethane (Tris), and acetic acid (all analytical grade) were obtained from Merck (Darmstadt, Germany). 5-Bromo-4-chloro-3-indolyl phosphate (BCIP), nitro blue tetrazolium (NBT), and pure nitrocellulose membrane (pore size 0.45 μm) were purchased from Bio-Rad Laboratories (Hercules, CA, USA). Boric acid (pro analysis) and dimethyl sulfoxide (DMSO, pro analysis) were from Fluka (Buchs, Switzerland). Dy-649P1 NHS-ester (λ_ex/em_ = 655/676 nm in ethanol according to the manufacturer) for fluorescence (FL) labeling was obtained from Dyomics (Jena, Germany). A 2.5 mM stock solution of the dye in DMSO was prepared for labeling. Further dilutions of the dye were performed applying only DMSO. For all solutions, water of Millipore grade (18.2 MΩcm resistivity at 25 °C) from a Simplicity UV water purification system (Millipore, Molsheim, France) was used throughout the entire investigation. Prior to application, all electrolytes were filtered with 0.2 μm pore size syringe filters (sterile, surfactant-free cellulose acetate membrane; Sartorius, Goettingen, Germany).

### Buffers and Sample Preparation

For nES GEMMA analysis, lectins and glycoproteins were dissolved in 20 mM NH_4_OAc pH 4.8 or 7.4 adjusted with acetic acid or ammonium hydroxide, respectively. Owing to the requirement of removal of nonvolatile salts (ConA, A1AT, and β-Gal solutions) 10 kDa cutoff spin filters (polyethersulfone (PES) membrane; VWR, Vienna, Austria) were used according to the manufacturer’s protocol. All analytes (direct solution or retentate) were then diluted to the required concentration (5–320 μg/mL). They were measured either directly or after 1 h incubation at 24 °C and 650 rpm for interaction experiments.

In the case of CE-on-a-chip experiments, analytes had to be FL labeled prior to electrophoresis. Thus, 150 μg protein (15 μg in the case of β-Gal) in 100 mM sodium borate pH 8.3 were mixed with 5 μM dye and incubated overnight in the dark at room temperature. Nonreacted dye was subsequently removed in the same way as described for the desalting step. Analyte concentrations were adjusted to 50–250 μg/mL with sodium borate prior to analysis. Analytes were either measured directly or after 1 h incubation of lectin and glycoprotein at 24 °C.

### nES GEMMA

nES GEMMA experiments were carried out on a system consisting of a model 3480 electrospray aerosol generator including a ^210^Po source, a model 3080 electrostatic classifier containing a nDMA unit, and a n-butanol driven model 3025A ultrafine CPC from TSI Inc. (Shoreview, MN, USA).

For operation in detection mode, the nDMA sheath flow was set to 15 liters per minute (Lpm; particle separation size range 2.0–64.4 nm EMD), for sampling a flow of 14 Lpm (2.0–67.3 nm EMD) was used. Samples were introduced via a 25 cm long cone-tipped fused silica capillary with an inner and outer diameter of 40 and 150 μm, respectively; 4 psid (pounds per square inch differential, approximately 0.3 bar) of pressure were applied to the sample vial for analyte introduction to the nES capillary in detection mode, whereas 2 psid were used for sampling. Higher pressure during long sampling experiments destabilized the spraying process and was thus avoided. The nES sheath gas (CO_2_ and filtered, dried air from a membrane dryer Superplus, Ludvik Industriegeräte, Vienna, Austria) was set to 0.6 Lpm and voltages were adjusted for a stable cone jet mode (2.0–2.5 kV). A median of 10 scans, 120 s each (100 s scan time, 20 s retrace time), yielded a spectrum (as shown in figures) and was used for data interpretation with the OriginPro software (v 9.1.0, OriginLab, Northampton, MA, USA).

For size-selected particle collections, a 3089 ENAS (TSI Inc.) replaced the CPC. The NC membrane was cut to 15 mm square. It was mounted on top of the center electrode using double-sided adhesive tape (Scotch/3 M, St. Paul, MN, USA), which was removed after sampling. The ENAS was operated at –9.5 kV and a gas flow rate of 1 Lpm. During collections of three times 12 h on three consecutive days about 475 μL of sample volume (20 μg/mL A1AT, a mixture of 10 and 20 μg/mL A1AT and SNA, respectively, or pure 20 mM NH_4_OAc, pH 7.4, as blank) were consumed.

### Capillary Electrophoresis-on-a-Chip

CE-on-a-chip was carried out on an Agilent 2100 Bioanalyzer platform (Waldbronn, Germany), a chip-based microfluidic system based on LIF detection (red diode laser, λ_ex/em_ = 635/685 nm). Owing to software modifications, large particles (e.g., viruses, protein complexes) are separated on chips originally designed for nucleic acid separation according to charge and size as previously described [34]. Briefly, the microfluidic channels were filled with 100 mM sodium borate pH 8.3 as background electrolyte (BGE) by applying pressure for 20 s using the Agilent Chip Priming Station. Twelve μL of BGE each were then applied to waste and buffer wells, and 6 μL of labeled analyte solutions to the sample wells. Prior to sample analysis on each chip, the separation channel was electrophoretically flushed with dye solution, 12.5 nM Dy-649P1 in BGE, followed by setup of the instruments optics and electrophoretic removal of the dye. Data were collected via the red laser of the instrument with the Agilent 2100 Expert software, exported, and plotted using the OriginPro software.

### Immunological Assay

After sample collection with nES GEMMA onto 0.45 μm NC membrane, the substrate was removed from the ENAS and tested for the presence of antitrypsin. Simultaneously, 10 and 50 ng of antitrypsin and SNA were directly applied to a control membrane and examined under the same conditions (dot blot assay). Both substrates were washed with TBS-Tween (20 mM Tris pH 8.3, 154 mM NaCl, 0.1% Tween 20) for 30 min and incubated overnight with anti-α_1_-antitrypsin antibody (1:9000, v:v, in TBS-Tween). They were washed three times in TBS-Tween for 5 min each, followed by an incubation with the anti-rabbit antibody conjugated to alkaline phosphatase (1:10,000, v:v, in TBS-Tween). The washing steps were repeated and the membranes further washed with TBS without Tween for 5 min. For color visualization, BCIP and NBT were prepared according to the manufacturer’s instructions and used for a 15 min incubation step. The reaction was stopped by addition of water.

### MALDI-MS

Experiments were performed on the MALDI-TOF-MS AXIMA TOF^2^ and, in the case of β-Gal, on the AXIMA-CFR *plus* instrument (Shimadzu Kratos Analytical, Manchester, UK) both equipped with nitrogen laser (λ = 337 nm). Both instruments were operated in linear positive ion mode. Samples were prepared on stainless steel MALDI target plates using the dried-droplet technique. Glycoprotein and β-Gal samples were applied 1:1 (v:v) ratio with 10 mg/mL SA in 0.1% TFA/ACN (1:1, v:v) as MALDI-MS matrix to a final amount of 10–20 and 1.5 pmol, respectively, on target and dried at room temperature.

## Results and Discussion

### Individual nES GEMMA Analysis of Glycoproteins and Lectins

For determination of the EMD, each analyte was measured individually with nES GEMMA at different concentrations in 20 mM NH_4_OAc (pH 7.4). The chosen buffer system should (1) be volatile, (2) resemble physiological conditions for glycoprotein–lectin interactions, and (3) be appropriate for a stable electrospray process. For experiments including lectins, higher NH_4_OAc concentrations destabilized the Taylor cone at the nES capillary tip and were consequently avoided.

**Figure 1:**
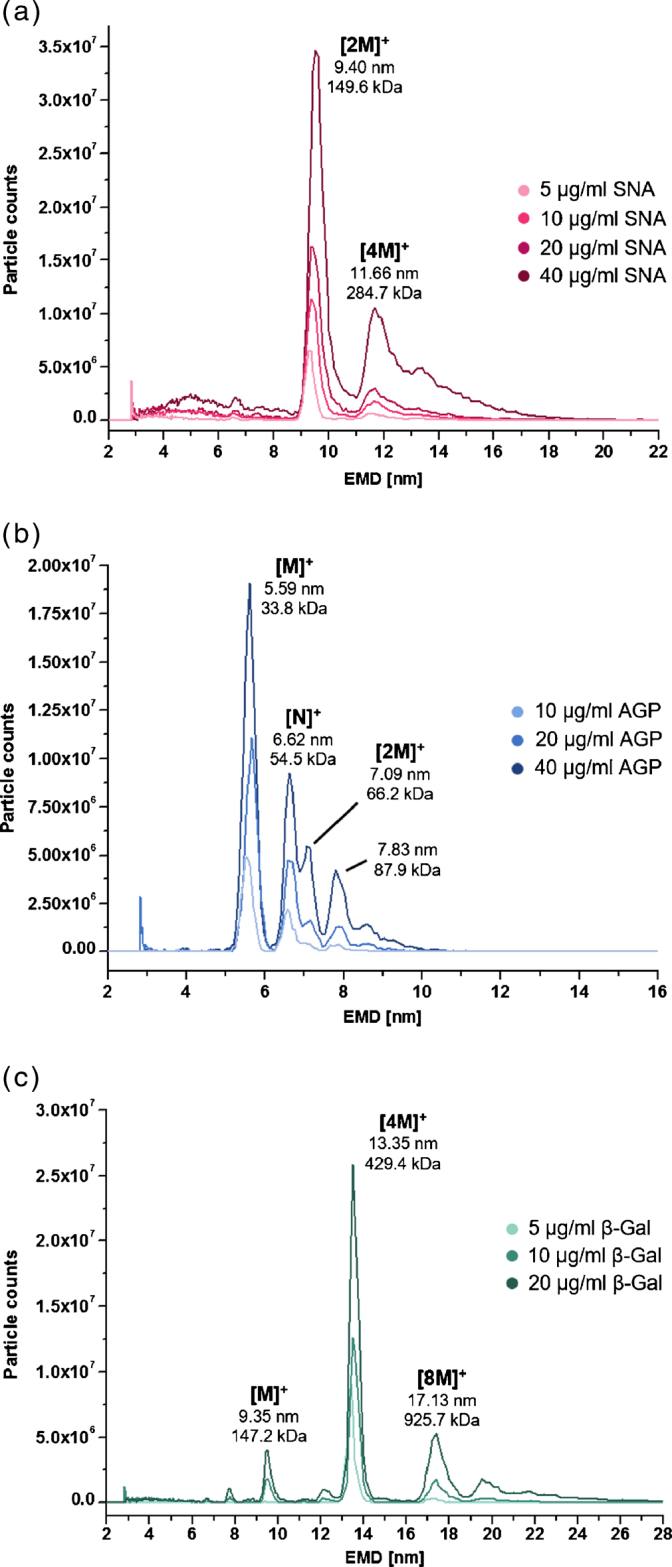
nES GEMMA analysis of different concentrations of the lectin SNA **(a)**, the glycoprotein AGP **(b)**, and the nonglycosylated β-Gal **(c)**. The subunits A and B of SNA are presented as M (M = AB) **(a)**. [N]^+^ represents a second constituent of AGP **(b)**

Figure 1 exemplarily displays the nES GEMMA spectra of the lectin SNA, the glycoprotein AGP, and the nonglycosylated protein β-Gal employed as negative control. For nES GEMMA spectra of the glycoproteins A1AT and Tf, as well as of the lectins WGA and SNA refer to the Supplementary Information. Figure 1a shows a dominating singly charged peak [2 M]^+^ representing a dimer of SNA with an EMD of 9.40 ± 0.09 nm, which corresponds to a MW of 149.6 ± 4.4 kDa calculated from an EMD/MW correlation [4]. This value is slightly deviating from the MALDI-MS derived MW of 130.1 ± 0.7 kDa (Table 1). SNA consists of four subunits (two of each identical; 2AB) held together by intramolecular disulfide bridges [35]. Owing to structure flexibilities of this complex in gas phase, the protein might appear bigger in nES GEMMA experiments with a higher MW calculated than measured with MALDI MS. Additionally, the singly charged tetramer [4 M]^+^ can be observed, which is especially apparent at higher concentrations. With increasing concentrations more than one analyte can be statistically present in a sprayed droplet, which leads to the formation of nonspecific gas-phase singly charged oligomers formed during the nES process [2]. These artificial oligomers can be distinguished from naturally formed biospecific complexes by a rapid loss of signal intensity or even disappearance with decreasing concentrations. Yet, lectins have a high tendency to aggregate. The fact that the tetramer signal did not completely vanish even at low concentrations points to biologically relevant tetramer formation already in solution.

In contrast, oligomer formation in the case of glycoproteins AGP (Figure 1b) was merely concentration-dependent and, hence, nES-induced. Furthermore, the existence of several AGP species with the most abundant one at 5.59 ± 0.05 nm (33.8 ± 0.9 kDa) was confirmed. These results were in good accordance to MALDI-MS data having, however, slightly higher values. Tf showed also gas-phase oligomerization (Supplementary Figure 1a) and A1AT likewise consisted of several species (Supplementary Figure 1b). β-Gal, on the other hand, a tetramer consisting of four identical, noncovalently linked 116 kDa subunits [31], showed only a less intensive peak of the monomer (9.35 ± 0.00 nm, calculated 147.2 ± 0.0 kDa). The detection of a high abundant tetrameric species (13.35 ± 0.06 nm, calculated 429.4 ± 5.7 kDa) demonstrated the instrument’s ability to keep noncovalent oligomers intact during analysis. In comparison, MALDI-MS revealed a MW of the monomeric species of 116.4 ± 0.1 kDa with the applied matrix. Table 1 summarizes the data for all investigated (glycol)proteins and lectins.

In accordance to previously published data on glycoprotein analysis by microchip capillary gel electrophoresis (MCGE) and SDS-PAGE [36], increasing glycan content led to signal broadening in MALDI-MS (Supplementary Figure S2). In contrast, the degree of glycosylation did not affect peak width or MW determination for nES GEMMA. This is in favor of analysis, high reproducibility of EMD values with deviations ≤ 1%, and small peak width (FWHM below 0.34 nm for all glycoproteins). However, this fact can also be considered as disadvantageous in regard to a loss of information about the glycosylation degree itself. Instead, FWHM values of peaks from nES GEMMA spectra were rather influenced by increasing EMDs (Table 1). In summary, gas-phase electrophoresis offers to be a reliable (±1%–5% mass accuracy for 8 kDa–1MDa proteins and protein complexes; reproducibility mostly better than ±0.1 nm) [37], sensitive (attomole amounts total consumption) [2], and fast (120 s per scan) alternative for glycoprotein analysis.

**Figure 2:**
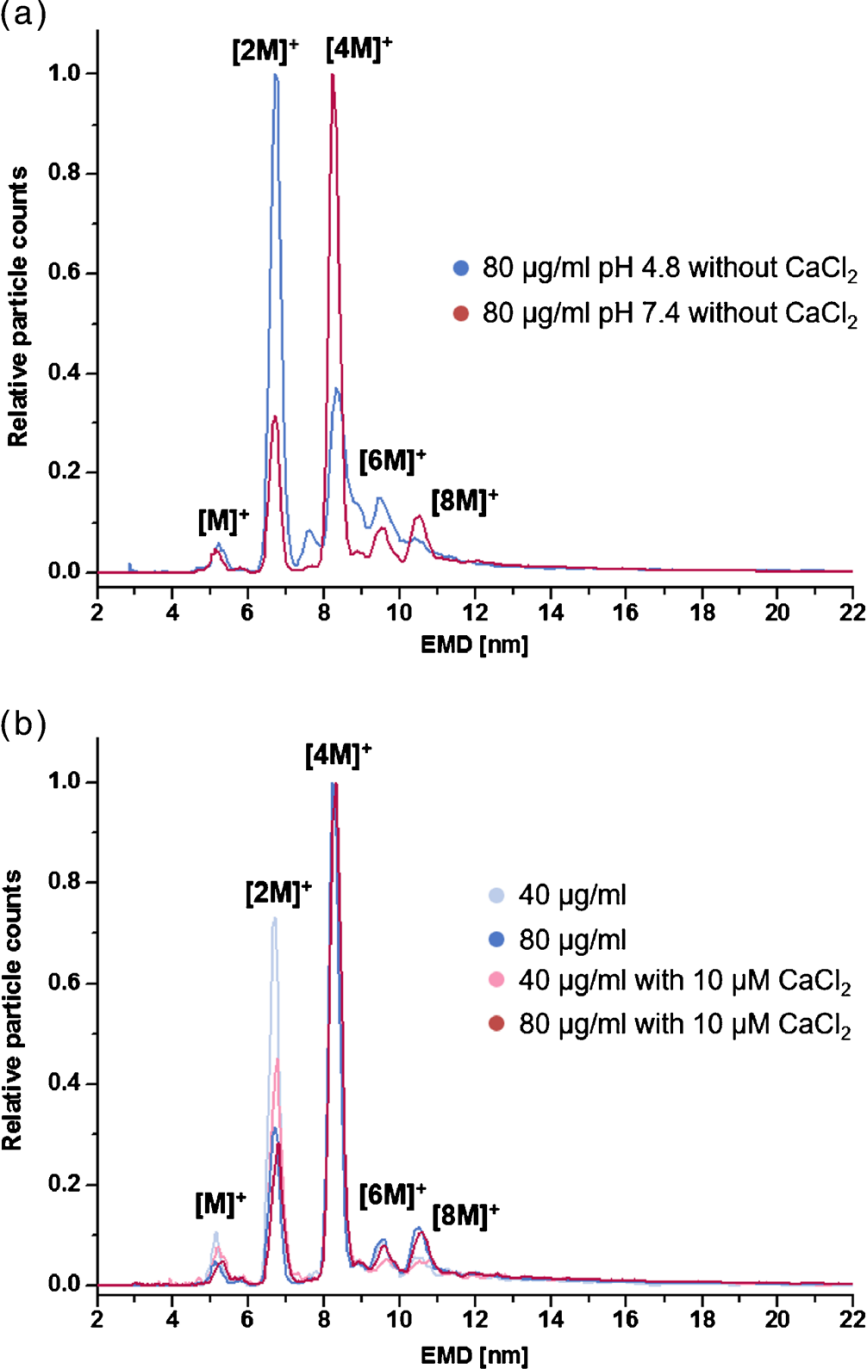
nES GEMMA analysis of the lectin ConA at different pH values **(a)** and at pH 7.4 with addition of 10 μM CaCl_2_ **(b)** in regard to oligomerization. ConA tetramers [4 M]^+^ are the biological dominant form

The nES GEMMA spectra of the other two lectins, WGA und ConA, in contrast, were more complex. The WGA spectrum was composed of several components, and ConA showed the formation of many oligomers (Supplementary Figure S1). In addition, the latter proved itself to be rather challenging during analysis because its high degree of oligomerization contributed to capillary clogging. This oligomerization is known to be pH sensitive: at ≤ pH 5 the lectin forms predominantly dimers and at pH ≥ 7 it primarily exists as tetramer [25]. This could also be shown by nES GEMMA (Figure 2a). At pH 4.8 mostly the dimeric form with only a small amount of tetrameric species could be observed. Those ratios were reverted at physiological pH. Next to ConA only β-Gal was affected by pH, which was unstable and not measurable from the acidic electrolyte.

Also known from literature is the fact that ConA requires the presence of divalent cations, e.g., calcium (Ca^2+^), for correct folding and carbohydrate recognition [25]. However, since high salt concentrations can lead to uncontrolled cluster formation in nES GEMMA [38], different CaCl_2_ concentrations were tested. No interferences were detected up to 10 μM CaCl_2_ in NH_4_OAc at pH 7.4 (data not shown). The salt addition stabilized the formation of biologically dominant ConA tetramers at low lectin concentrations and was thus considered as appropriate for ConA interaction studies with glycoproteins (Figure 2b). At higher CaCl_2_ concentrations, measurements of ConA were not feasible and, therefore, an additional influence of CaCl_2_ not investigable.

### nES GEMMA Interaction Analysis of the Lectins with Glycoproteins

In order to investigate the interaction of SNA, ConA, and WGA with the glycoproteins, the lectins were incubated with each glycoprotein separately at different concentrations and subsequently analyzed with nES GEMMA. Additionally, experiments were carried out with β-Gal as a nonglycosylated negative control.

Owing to the fact that all in this study using glycoproteins showed various degrees of sialylation, a recognition by SNA was expected to be positive in all cases but with different affinities and, i.e., various intensities. Keeping the glycoprotein concentration constant during measurements and increasing only the amount of lectin, a steady decrease of the glycoprotein signal hints the formation of the biospecific complex with SNA. The emerging complex is expected to be detected at the respective EMD (EMD_calculated_), which can be calculated from the sum of the individual MWs and the given EMD / MW correlation [4]. Furthermore, data (EMD/MW_experimental_) can be compared with theoretical values for the MW_calculated_ of the lectin–glycoprotein complex. A close agreement of both values confirms the detection of the non-covalent complex.

**Figure 3:**
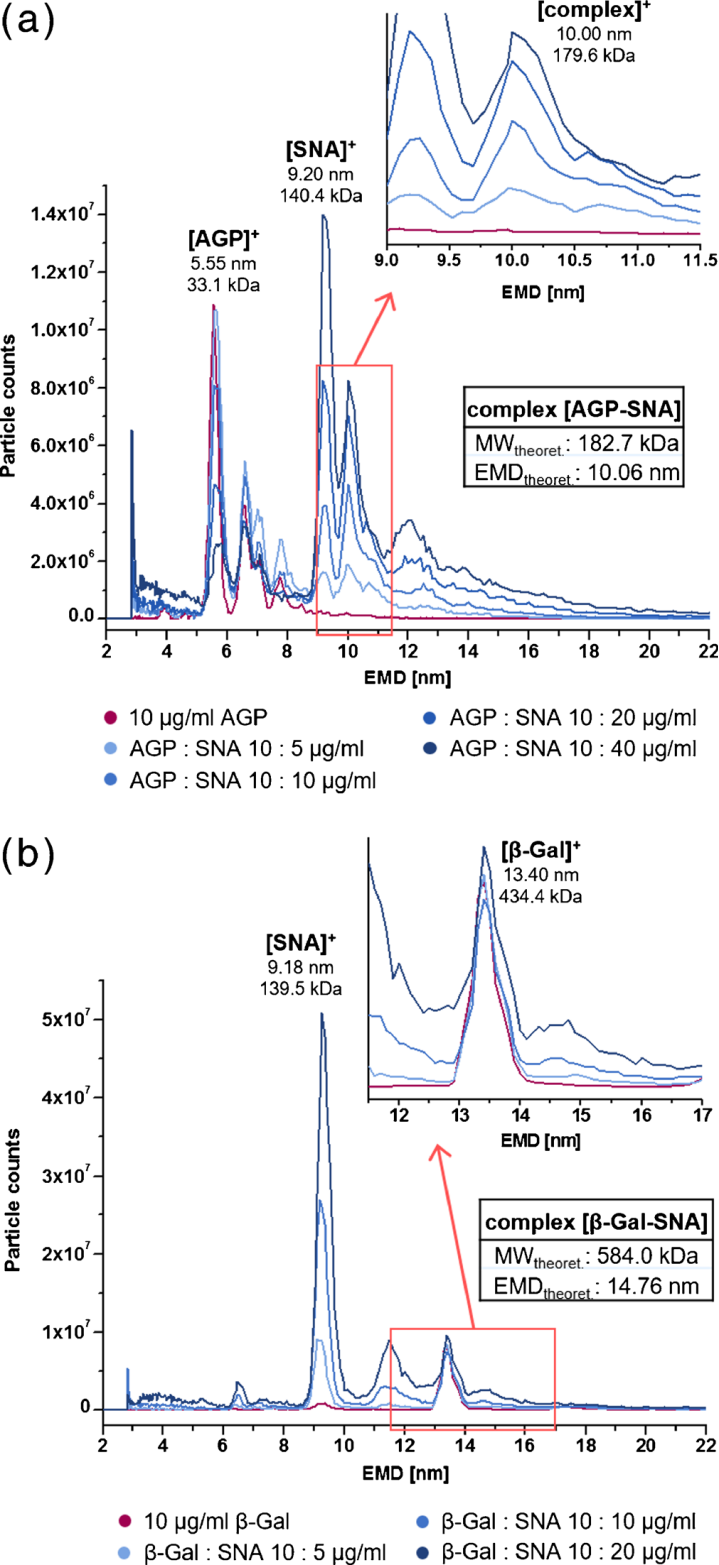
nES GEMMA analysis of AGP **(a)** or β-Gal as negative control **(b)** incubated with different concentrations of SNA

Figure 3a presents the incubation of SNA with AGP, which has the highest degree of sialylation. As expected, the intensity of the monomeric AGP signal at 5.55 nm decreased by 75% with increasing SNA concentrations. Moreover, the biospecific complex at 10.06 nm EMD could clearly be detected. In comparison, no according signals were observed for interactions of SNA with the nonglycosylated β-Gal (complex expected at 14.76 nm EMD, Figure 3b). This proved for the first time the capability of nES GEMMA to detect specific lectin-glycoprotein bindings, bindings that are rather weak and, therefore, difficult to analyze (dissociation constants in the mM to high nM range, antibody-epitope bindings are 100- to 1000-fold stronger).

Similar results as with AGP could be gained during the incubations of SNA and A1AT (Supplementary Figure S3a). For A1AT also the SNA concentration was kept constant while steadily increasing the amount of A1AT. Results were the same; the expected signal of the noncovalent complex was observed while the SNA peak decreased (Supplementary Figure S3b). The analysis of the interaction of Tf with the lectin SNA led to comparable findings (Supplementary Figure S3c). However, contrary to AGP and A1AT, the signal for the complex was not as distinct and exhibited lower signal intensities. From this, a lower binding specificity of SNA towards Tf could be concluded, which is in agreement with the comparably lower degree of sialylation. From these findings, we conclude that nES GEMMA can distinguish different lectin binding strengths and specificities towards varying glycoproteins.

The interactions of ConA and WGA with each glycoprotein and β-Gal were additionally investigated to get a more profound understanding of nES GEMMA capabilities (for exemplary results, see Supplementary Figure S4). In the case of ConA, a direct detection of the complex signals was significantly impeded by the lectin’s own oligomer peaks, which overlaid the expected glycoprotein–ConA complex. Nevertheless, the decrease of the glycoprotein signals could be observed and used as an indicator for a positive binding: the Tf peak showed the greatest reduction followed by AGP, whereas the A1AT peak diminished only slightly. Also the β-Gal signal decreased slightly, which hinted to minor unspecific interaction between the nonglycosylated protein and ConA.

Investigating glycoprotein interactions with WGA turned out to be rather challenging. Owing to similar MWs of the lectin monomers/oligomers with the glycoproteins, the lectin signals did not only overlay the lectin–glycoprotein complex peaks but also those from the glycoproteins. Therefore, neither the decrease in glycoprotein signal nor the newly formed complex signal could be observed. Enhanced resolution is expected for instruments having higher sheath flow rates (e.g., the second generation MacroIMS device from TSI Inc., PDMA [39, 40], or a Vienna type DMA [41]) allowing, then, hopefully for improved signal separation. As a consequence of these findings, additional investigations concentrated on SNA, which showed the most convincing results so far.

### Interaction Analysis of SNA by Means of CE-on-a-Chip Experiments

**Figure 4:**
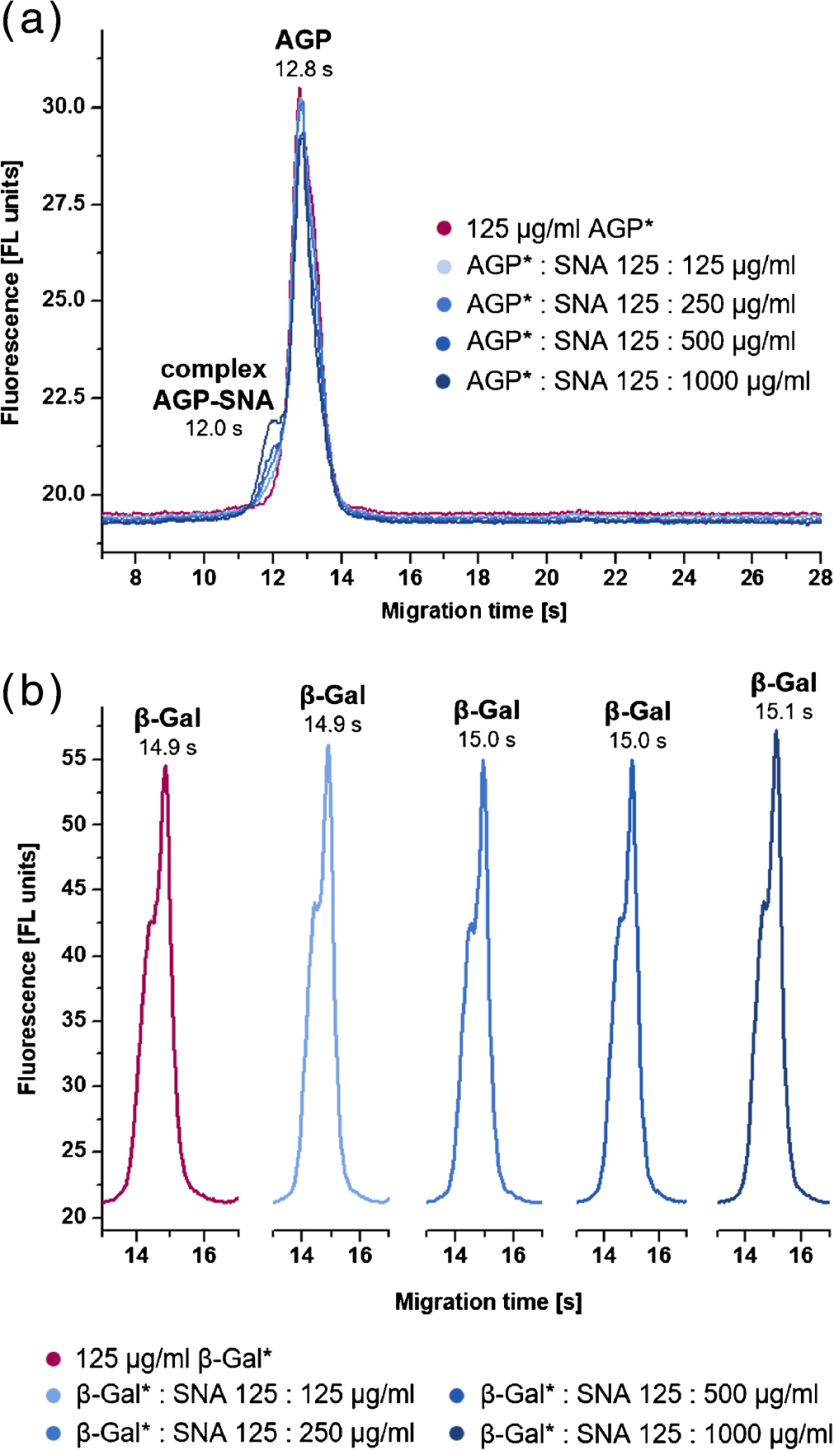
CE-on-a-chip analysis of SNA with AGP and β-Gal: electropherograms of incubations of AGP **(a)** and β-Gal **(b)** with increasing concentrations of unlabeled SNA, respectively. Labeled proteins are marked with an asterisk (*)

For confirmation of nES GEMMA results, the formation of biospecific lectin–glycoprotein complexes was additionally examined by CE-on-a-chip, a liquid-phase based chip electrophoresis system. Fluorescence labeled glycoproteins and the nonglycosylated β-Gal were incubated with different concentrations of unlabeled SNA. As with nES GEMMA, the formation of a new interaction-relevant signal and the decrease of the glycoprotein peak were expected for rising SNA concentrations. Figure 4a shows the slightly declining signal of AGP with rising SNA content and the clearly emerging glycoprotein-lectin peak at 12.0 s. The negative control β-Gal repeatedly showed no interaction with SNA, maintaining a constant migration pattern despite increasing SNA concentrations (Figure 4b). For A1AT a decrease of signal intensity was observed, whereas the signal for the complex was growing significantly (Supplementary Figure S5a). In addition, it became obvious that the SNA–A1AT complex exhibited the same migration time as a for us today unknown constituent of A1AT (marked with an asterisk in Supplementary Figure 5). The fact that at constant A1AT concentration the signal at 12.6 s showed up to six times increased intensities with rising SNA content allowed for the conclusion that this peak in fact is induced by the glycoprotein–lectin complex. The drastic change in the peak pattern of A1AT hinted a strong interaction with SNA, which was more explicit than with AGP. Tf interacted likewise stronger with SNA than AGP (Supplementary Figure S5b). Thus, all three glycoproteins proved to interact with SNA as already shown with nES GEMMA. Consequently, these experiments corroborated nES GEMMA findings. Reduced or altered binding between AGP and SNA, as detected with CE-on-a-chip, might result from covalently bound FL labels to glycoproteins. They can modify the protein structure and, therefore, influence the binding strength and specificity towards the lectin.

### Collection of the Biospecific Lectin–Glycoprotein Complex and Its Immunological Identification

SNA-A1AT complexes were collected after gas-phase size-separation with an ENAS on a NC membrane. After sampling the membrane was removed for subsequent immunologic analysis with colorimetric detection. The color formation on the membrane is based on an epitope recognition of the protein in its native conformation by the antibody. Therefore, it requires the preservation of the collected particles’ three-dimensional structure throughout the separation with nES GEMMA and collection process.

**Figure 5:**
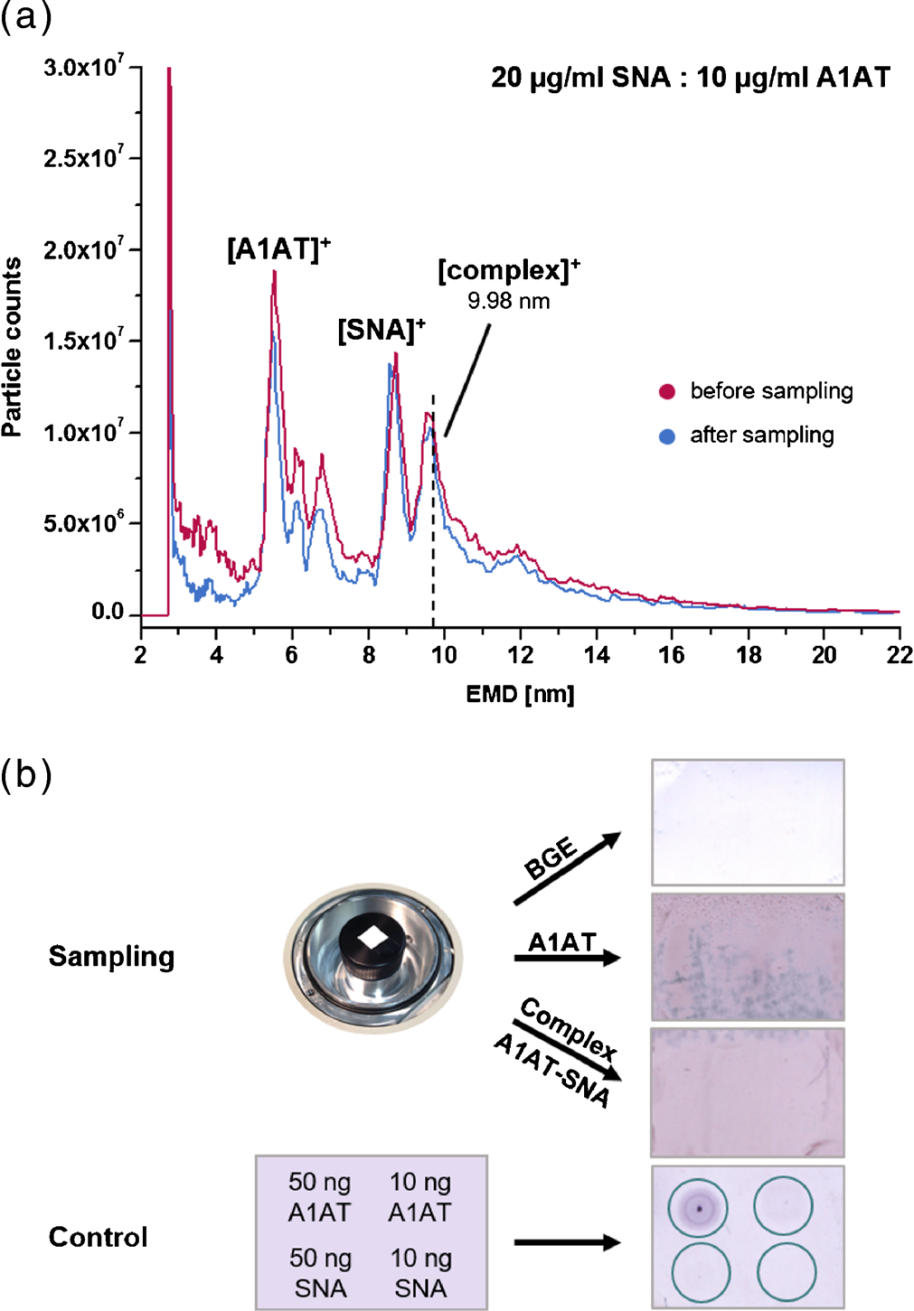
Collection of SNA–A1AT complexes using an ENAS (particle fraction collector). The complex was collected onto NC at 9.96–10.05 nm for 36 h on three consecutive days **(a)** exemplarily showing the sampling of 1 day) followed by immunological identification via color visualization in comparison to a control dot blot experiment **(b)**. For further verification, also pure BGE (9.98 nm) and A1AT (5.60–5.65 nm) were sampled onto NC membrane and immunologically examined **(b)**. The dotted line marks the EMD of sampling of the exemplary day **(a)**

By applying A1AT directly on the NC membrane, detection limits for the chosen dot blot assay down to 10 ng glycoprotein were revealed. Based on this, the necessary sampling time of about 36 h was calculated from the applied A1AT-SNA concentrations (10 and 20 ng/μl, respectively, Figure 5a and Supplementary Figure S6) and the injection rates (2 psid of applied pressure). For these 36 h we assumed that (1) less than 5% (usually about 1%) of the overall electrosprayed analytes are reduced to singly charged particles in the neutralizing chamber [42], (2) the sample is a mixture of A1AT, SNA, and A1AT–SNA complex, from which only the latter is of interest for analysis and, therefore, collected onto the NC membrane, (3) that at least 30% to 50% of the present A1AT is forming a complex with SNA, and (4) that no singly charged complex particle is lost during nDMA separation and NC collection. From this we expected about 20 ng glycoprotein–lectin complex to be finally collected on the NC, amounts sufficient for dot blot like analysis.

The glycoprotein–lectin complex was sampled at 9.96–10.05 nm EMD, and pure A1AT was collected at 5.60–5.65 nm EMD for immunologic analysis (Figure 5b). Additionally, the BGE was sprayed as a blank for 36 h and sampled at the respective EMDs. In order to verify that the dot blot analysis was specific for A1AT but not SNA or its oligomers, a control was carried out by direct application of SNA and A1AT on NC membranes. Only A1AT showed interaction, proving that any color formation was a direct correlation to A1AT presence. First, the preservation of the native conformation after gas-phase separation of A1AT alone was checked by staining the NC membrane after sampling, which could be observed visually compared with the BGE blank. We found that also the sampling of the SNA–A1AT complex onto the NC membrane showed a noticeable staining comparable to A1AT sample. Interestingly, no distinct spot in the size of the ENAS electrode (9.5 mm diameter) was found, as observed previously after collecting significantly larger particles [16]. In our case, the applied NC membrane was evenly stained, probably due to the fact that the ENAS voltage was not high enough to deviate the particles from their trajectory imposed by the high nDMA sheath flow and to focus them on a distinct area. An increase of the applied voltage could solve this problem and lead to a shorter sampling time as the analyte concentration would be increased on the NC membrane. However, due to instrument limitations, this approach cannot be realized at the moment.

## Conclusions

The nES GEMMA system is a promising platform for the analysis of lectin–glycoprotein interactions as shown in the given study for the first time. Especially, data interpretation is much easier for singly charged particles than ESI spectra of multiply charged noncovalent complexes (data deconvolution can be omitted). Furthermore, sample preparation is only dependent on the formation of a complex in NH_4_OAc at the appropriate pH. Today, noncovalent interaction studies are of utmost interest for a better understanding of biological interactions (as for example in molecular machines). nES GEMMA is a valuable tool to study lectin–glycoprotein interactions in regard to interaction specificities and binding strength. We found that the ambient setup of the instrument allowed for the detection of rather weak interactions, which are difficult to maintain in vacuum-based mass analyzers. Working under relatively soft conditions, nES GEMMA even enables sampling of these complexes in their biologically native form. For the first time, bionanoparticles in the rather low nm size range were collected by the ENAS device and analyzed by an immunologic assay. ENAS sampling corroborated correct peak assignment of the noncovalent complex consisting of the lectin SNA and the glycoprotein A1AT in mixed samples. Moreover, it showed the maintenance of interactions formed in liquid phase during gas-phase separation without affecting the native state of the complex. This finally confirms that nES GEMMA allows for separation and detection of biospecific, noncovalent complexes (but relatively weak compared with virus-antibody or virus-like particle-antibody fragment complexes), as well as their successful sampling for further analyses.

## Electronic supplementary material

Below is the link to the electronic supplementary material.

ESM 1 (DOCX 2296 kb)
