# Supplementary material for: nES GEMMA Analysis of Lectins and Their Interactions with Glycoproteins – Separation, Detection, and Sampling of Noncovalent Biospecific Complexes
Source: J Am Soc Mass Spectrom. 2016 Sep 19;28(1):77–86. doi: 10.1007/s13361-016-1483-0 (PMC5174143; doi:10.1007/s13361-016-1483-0)
Supplement: Supplementary file 1 — (DOCX 2296 kb) [file 13361_2016_1483_MOESM1_ESM.docx]

**Supplementary Material**

**nES GEMMA Analysis of Lectins and their Interactions with Glycoproteins – Separation, Detection, and Collection of the Non-covalent Biospecific Complexes**

**[Running Title: nES GEMMA of Lectin-Glycoprotein Complexes]**

Nicole Y. Engel, Victor U. Weiss, Martina Marchetti-Deschmann, Günter Allmaier

Institute of Chemical Technologies and Analytics, TU Wien, Vienna, Austria

**Address reprint requests to**:

Prof. Günter Allmaier, Institute of Chemical Technologies and Analytics, TU Wien (Vienna University of Technology), Getreidemarkt 9/164-IAC, A-1060 Vienna, Austria

**E-mail:** guenter.allmaier@tuwien.ac.at

**Phone:** +43 - 1 - 58801 - 15160

**Fax:** +43 - 1 - 58801 - 15199


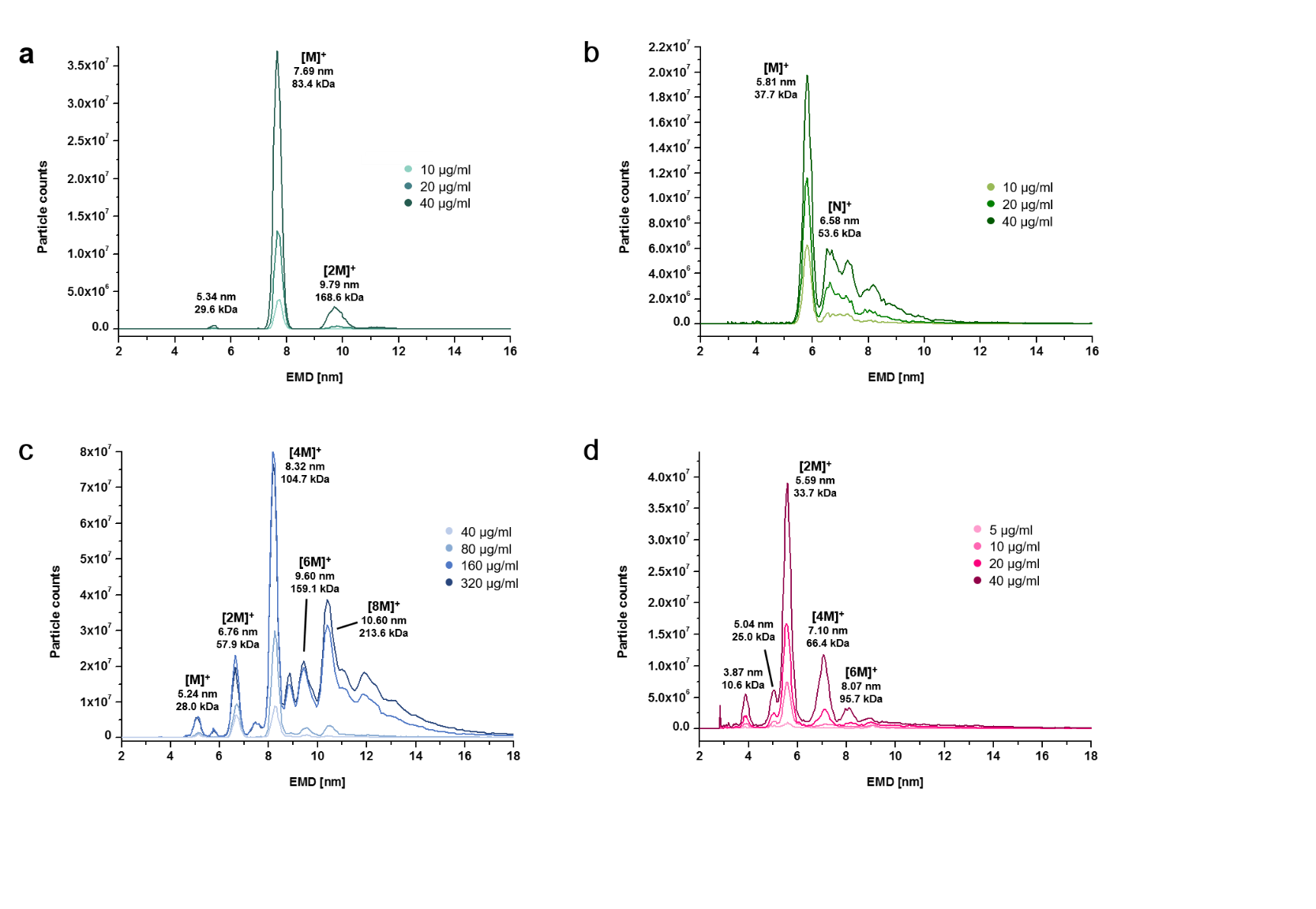


**Supplementary Figure 1.** nES GEMMA analysis of different concentrations of the glycoproteins Tf (**a**) and A1AT (**b**), as well as the lectins ConA (**c**) and WGA (**d**). [N]^+^ represents a second constituent of A1AT (**b**).


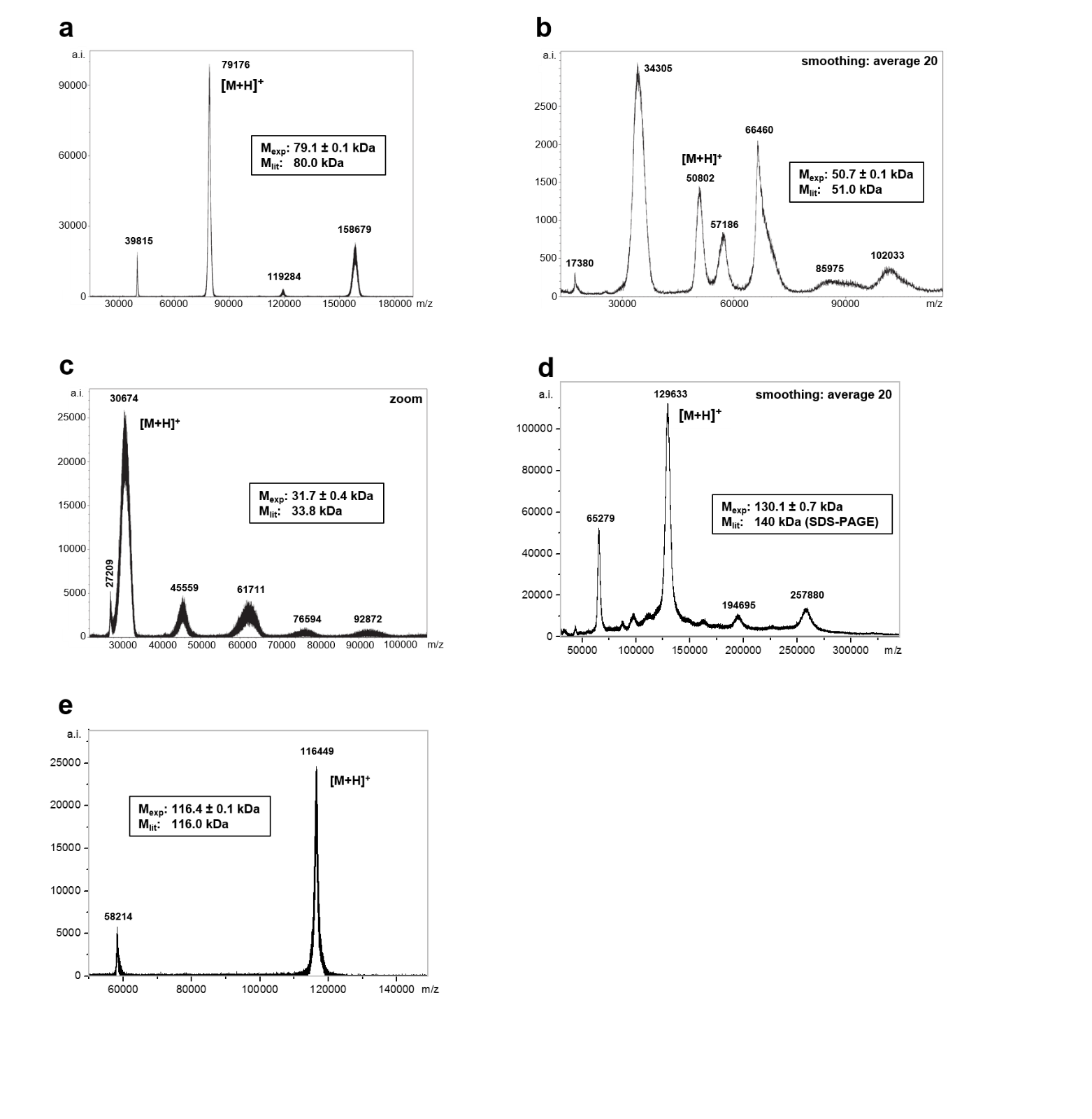


**Supplementary Figure 2.** Positive ion MALDI mass spectra of 10 pmol Tf (**a**), 10 pmol A1AT (**b**), 10 pmol AGP (**c**), 20 pmol SNA (**d**), and 1.5 pmol β-Gal (**e**) on target.


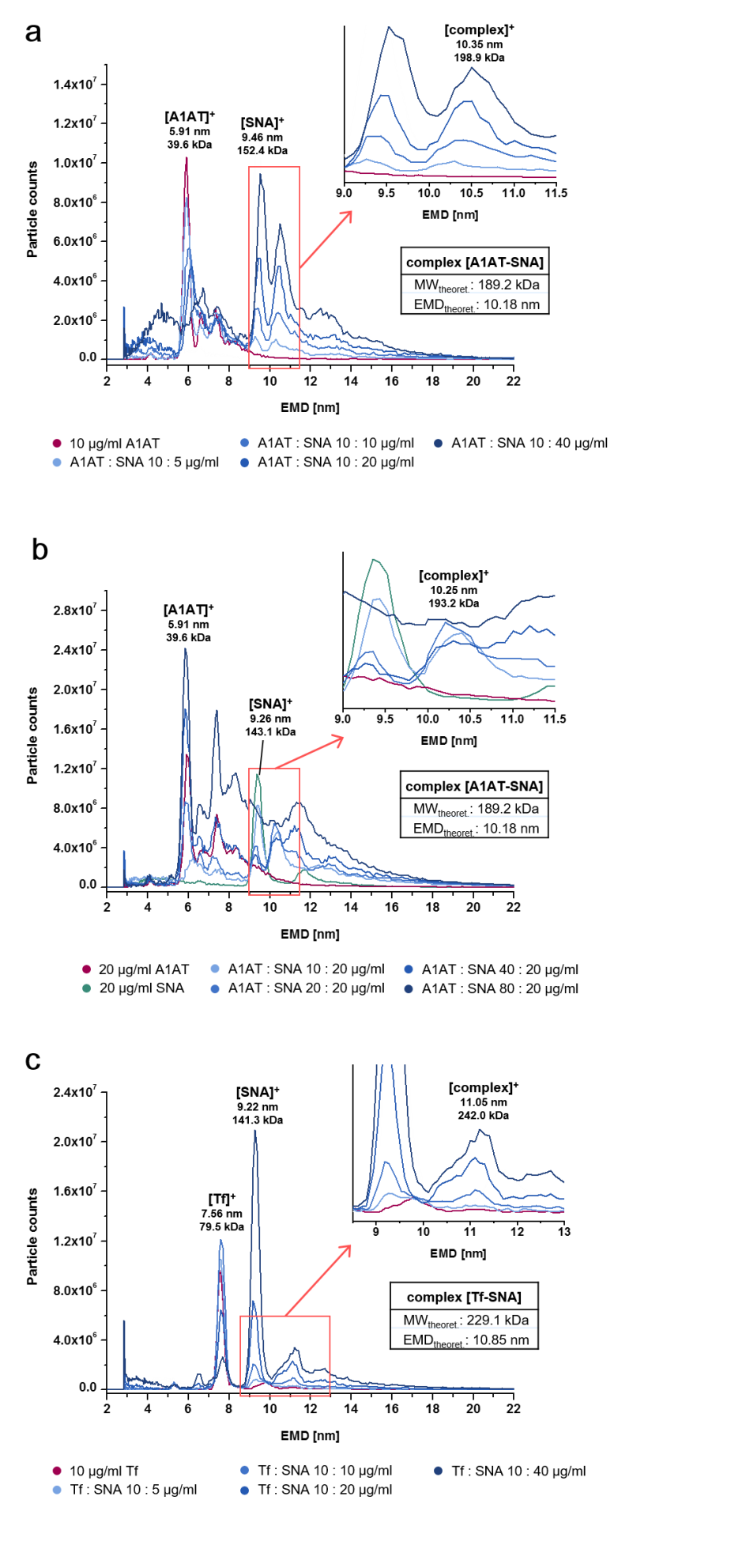


**Supplementary Figure 3.** nES GEMMA analysis of A1AT (**a**) and Tf (**c**) incubated with different concentrations of SNA, as well as SNA incubated with different concentrations of A1AT (**b**).


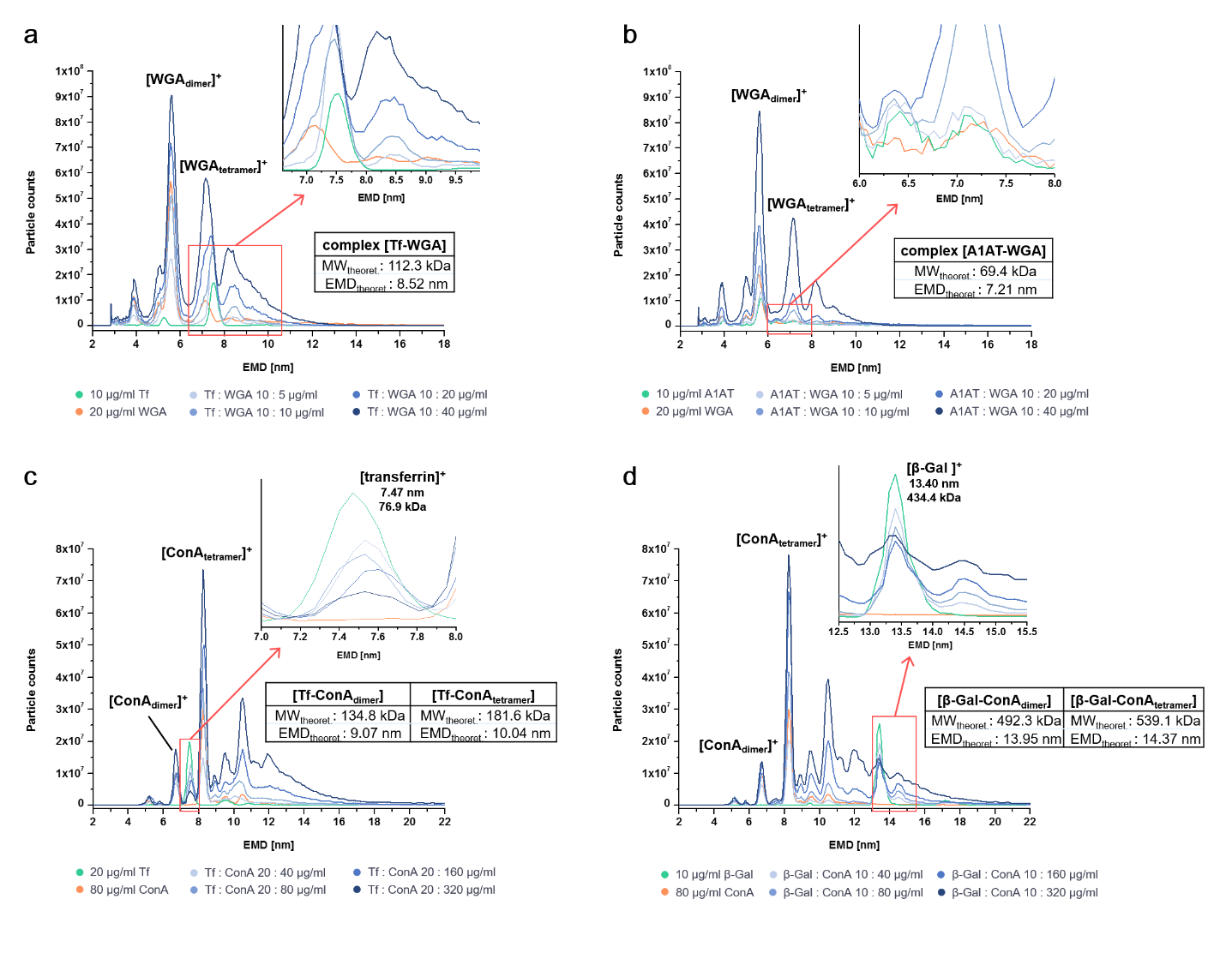


**Supplementary Figure 4.** nES GEMMA analysis of different concentrations of WGA incubated with the glycoproteins Tf (**a**) and A1AT (**b**) and of ConA incubated with Tf (**c**) and β-Gal as negative control (**d**).


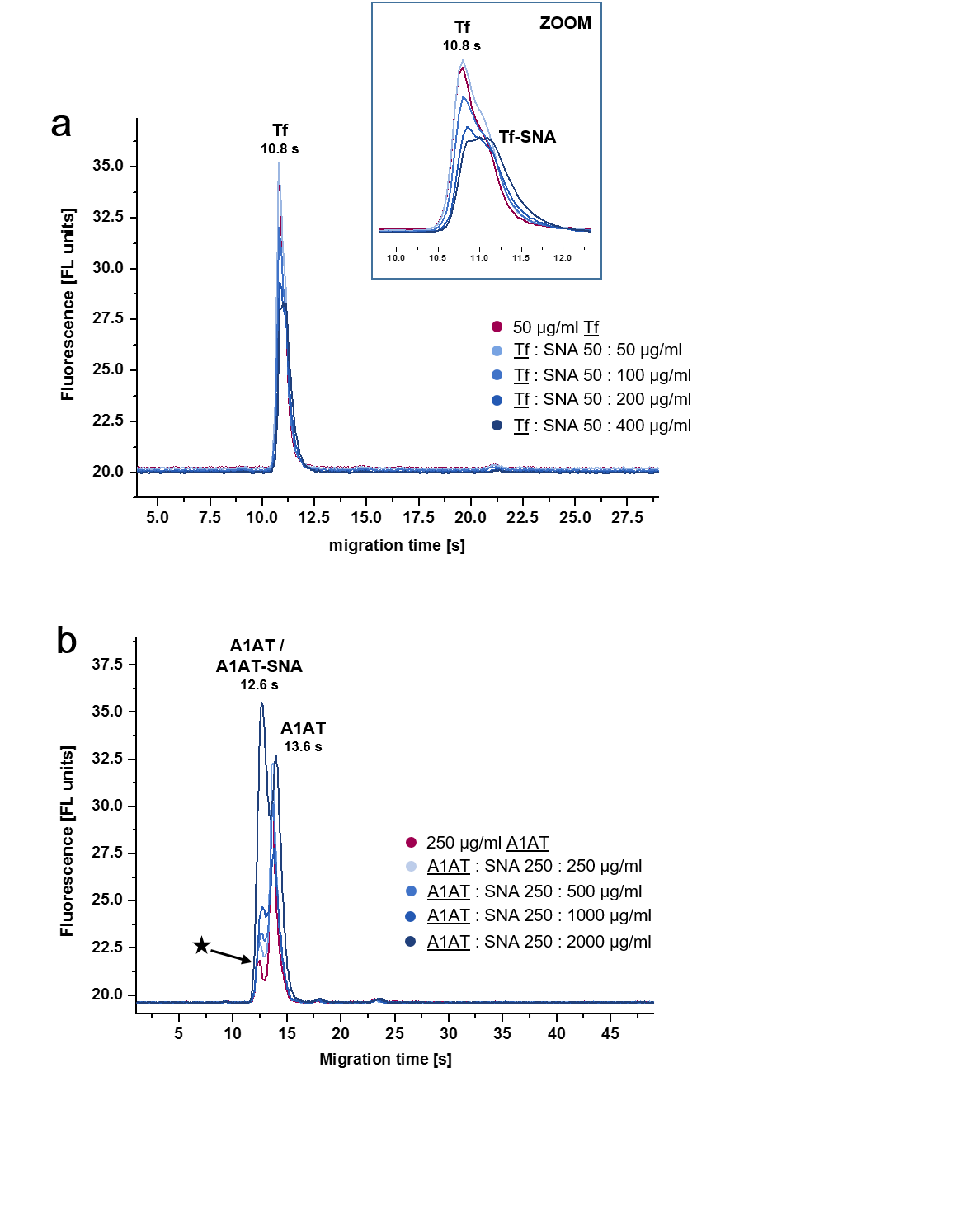


**Supplementary Figure 5.** CE-on-a-chip interaction analysis of labeled Tf (**a**) and A1AT (**b**) with rising concentrations of unlabeled SNA. Labeled proteins are underlined. An unknown constituent of A1AT is marked with an asterisk (*).


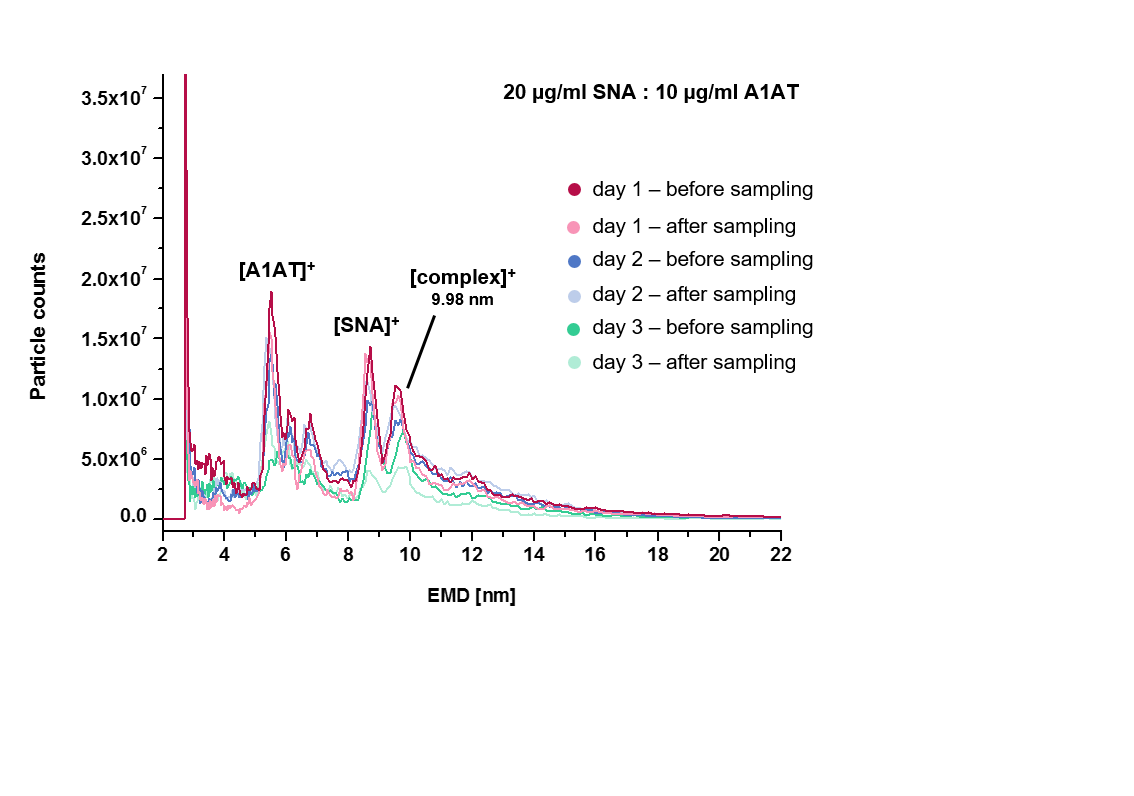


**Supplementary Figure 6.** Sampling of SNA-A1AT complexes onto NC at 9.96 – 10.05 nm for 36 h on three consecutive days using an aerosol sampler.
